# A study of the factors associated with cervical spinal disc degeneration, with a focus on bone metabolism and amino acids, in the Japanese population: a cross sectional study

**DOI:** 10.1186/s12891-018-2055-1

**Published:** 2018-05-17

**Authors:** Kanichiro Wada, Toshihiro Tanaka, Gentaro Kumagai, Hitoshi Kudo, Toru Asari, Daisuke Chiba, Seiya Ota, Keita Kamei, On Takeda, Shigeyuki Nakaji, Yasuyuki Ishibashi

**Affiliations:** 1 Department of Orthopedic Surgery, Hirosaki University Graduate School of Medicine, Zaifu-cho 5, Hirosaki, Aomori 036-8562 Japan; 2 Department of Social Health, Hirosaki University Graduate School of Medicine, Zaifu-cho 5, Hirosaki, Aomori 036-8562 Japan

**Keywords:** Cervical spine, Disc degeneration, Bone metabolism, Amino acid

## Abstract

**Background:**

The physical and biochemical factors responsible for cervical disc degeneration, and resulting in various spinal disorders, remain unclear. This study aimed to evaluate the correlation between cervical spinal canal stenosis and degeneration of intervertebral discs, and to analyze the factors related to disc degeneration in the Japanese population.

**Methods:**

Three hundred and forty-four Japanese general residents underwent investigations, including magnetic resonance imaging of the cervical spine, in our health check project. We measured anteroposterior diameters at the levels of the cervical spinal disc in mid sagittal plane magnetic resonance imaging and evaluated disc degeneration. Spearman correlation coefficient was used to evaluate whether the diameters were correlated with disc degenerative scores. Stepwise multiple linear regression analysis was conducted with the score of disc degeneration as the dependent variable; and age, physical measurement values, bone mineral density of the forearm, and the value of serum bone metabolic markers and amino acids as the independent variables for each sex.

**Results:**

As the age increased, the anteroposterior diameters decreased in both sexes. The minimum anteroposterior diameters were correlated with the disc degenerative scores (Spearman *r* = − 0.59, *p* < 0.001 in men, Spearman *r* = − 0.53, *p* < 0.001 in women). In multiple linear regression analysis, age, cross-linked N-telopeptide of type 1 collagen and isoleucine were significantly correlated with the cervical disc degenerative score in men (R^2^ = 0.47), and age and lysine were significantly correlated with the degenerative score in women (R^2^ = 0.50).

**Conclusion:**

The factors responsible for cervical disc degeneration differed between men and women. Whether modifying these significant factors is possible, or whether this intervention would contribute to prevention of disc degeneration requires future studies.

## Background

Cervical myelopathy is a common degenerative spinal disease affecting the activities of daily living [1–3]. One of the pathophysiologies of cervical myelopathy is cervical spinal cord compression, which might be secondary to spinal canal stenosis. Causes of cervical spinal canal stenosis include developmental canal stenosis, intervertebral disc protrusion into the spinal canal, and thickening of the ligamentum flavum [4]. Some cervical degenerative changes are more pronounced in the intervertebral discs, and these changes progress with aging [5, 6]. In some epidemiologic studies, the prevalence of cervical canal stenosis increased with aging [7, 8].

Risk factors of intervertebral disc degeneration are reported to be age [6, 9], genetic factors [10], smoking [11], labors [12, 13], and lumbar spine disorders [14, 15]. Although paraspinal muscle atrophy was reported to be associated with lumbar disc degeneration [16, 17], no association between cervical muscle and disc degeneration was reported [18]. The relationship between trunk/extremity muscles and cervical disc degeneration remains unclear.

With regards to the biochemical markers of musculoskeletal disorders, some bone metabolism markers were associated with lumbar disc narrowing [19]. One of the useful biomarkers related to cartilage turnover, that might predict progressive knee osteoarthritis, is serum hyaluronic acid which leaks into the blood from degenerated cartilage [20]. Amino acids are chemical constituents of the intervertebral disc [21]; however, there are only a few reports about useful biomarkers for prediction of cervical intervertebral degeneration.

The following issues remain unclear: (1) how are cervical spinal canal stenosis and intervertebral disc degeneration related in each level or overall, and (2) what are the physical features and biochemical makers related to cervical intervertebral degeneration.

The present cross-sectional study aimed to evaluate the correlation between cervical spinal canal stenosis and degeneration of the intervertebral disc, and to analyze whether physical and biochemical data (such as bone metabolism markers and amino acids) relate to cervical intervertebral disc degeneration in the general population.

## Methods

### Participants and outline of Iwaki Health Promotion Project

The Iwaki Health Promotion Project is a health check program designed to improve the average life span. This program was initiated in 2005, and has been conducted annually over a 10-year period. Approximately 1000 adults, aged 20 years and older, living in the Iwaki area of Hirosaki City, Japan, participate every year. In addition to orthopedic specialists, physicians, general surgeons, gynecologists, urologists, psychiatrists, dermatologists, and dentists are involved in this project. As one aspect of the multiple-focused check, we collect biochemical and biomechanical data related to spine disorders, including several parameters of bone metabolism.

A total of 1112 individuals (20–91 years old, 431 men and 681 women) participated in the Iwaki Health Promotion Project in 2015. All participants answered questionnaires about their past medical history, life style, fitness habits, occupational history, family history, and health-related quality of life. They also provided disease-specific information including a description of their cervical and extremity symptoms. They underwent physical measurements such as height, weight, individual body resistance, etc., and underwent physical examination for assessment of neurological status. In addition, bone mineral density and biochemical examination were performed to evaluate bone status. Participants with a history of stroke, cerebral bleeding, trauma or previous surgery of the cervical spine, systemic diseases involving the cervical spine (e.g., rheumatoid arthritis), and those who did not complete the questionnaires were excluded. Magnetic resonance imaging of the cervical spine was evaluated randomly by age. Finally, 344 people (20–86 years old, 151 men and 193 women) were included in our study (Table 1). The mean (±SD) ages of the male and female participants were 54.2 (±15.1) and 55.5 (±14.6) years, respectively.

**Table 1:** Demographic data

	Men	Women	*P* value
N total	151	193	
20–39 years	30	38	
40–49 years	28	31	
50–59 years	32	37	
60–69 years	33	47	
70- years	28	40	
Age (years)	54.3 ± 15.1	55.5 ± 14.6	0.502
BMI (kg/m^2^)	23.9 ± 3.3	22.2 ± 3.3	0.000
SMI (kg/m^2^)	18.9 ± 1.6	15.4 ± 1.5	0.000
T score of BMD (%)	100.0 ± 10.0	90.2 ± 16.0	0.000
BAP (μg/dL)	14.6 ± 4.6	14.9 ± 6.2	0.723
NTx (nMBCE/L)	18.5 ± 5.3	18.8 ± 5.9	0.833
TRACP-5b (mU/dL)	389.9 ± 124.1	419.9 ± 197.6	0.312
total P1NP (μg/L)	48.1 ± 16.3	55.2 ± 22.8	0.006
25(OH)D3 (ng/mL)	10.7 ± 7.0	16.2 ± 6.0	0.000
Pentosidine (nmol/mL)	28.0 ± 9.7	29.8 ± 12.9	0.224
Isoleuicine (nmol/mL)	70.4 ± 14.1	53.5 ± 8.7	0.000
Threonine (nmol/mL)	140.1 ± 35.5	129.8 ± 29.3	0.006
Tryptophan (nmol/mL)	52.7 ± 7.7	46.6 ± 6.3	0.000
Valine (nmol/mL)	238.3 ± 36.8	200.9 ± 30.8	0.000
Histidine (nmol/mL)	86.1 ± 9.6	81.1 ± 7.5	0.000
Phenylalanine (nmol/mL)	64.1 ± 10.8	58.2 ± 9.1	0.000
Methionine (nmol/mL)	29.0 ± 5.4	24.9 ± 3.7	0.000
Lysine (nmol/mL)	216.6 ± 33.5	193.8 ± 28.7	0.000
Leucine (nmol/mL)	132.9 ± 21.4	104.0 ± 15.2	0.000

*Abbreviations*: *BMI* body mass index, *SMI* skeletal muscle index, *BMD* bone mineral density, *BAP* bone-specific alkaline phosphatase, *NTx* cross-linked N-telopeptide of type 1 collagen, *TRACP-5b* bone tartrate-resistant acid phosphatase-5b, *total P1NP* total type 1 procollagen N-terminal propeptide

### Assessment of physical characteristics

Individual body resistance at 50 kHz (R) was measured using the Tanita MC-190 body composition analyzer (Tanita Co., Tokyo, Japan). Skeletal muscle mass was calculated using Janssen’s regression equation, which is based on the relationship between bioelectrical impedance analysis and skeletal muscle mass measured by magnetic resonance imaging [22]. Skeletal muscle mass (kg) = [(height^2^/R × 0.401) + (sex × 3–825) + (age × − 0.071)] + 5.102, where height is the height in centimeters; R is the resistance in ohms; for sex, men = 1 and women = 0; and age is in years. The coefficient of determination (R^2^) in this regression equation was 0.86; standard error values were 2.7 kg or 9%. Skeletal muscle index was calculated using skeletal muscle mass/height^2^ × 102 (kg/m^2^) for standardizing the differences influenced by height.

### Assessment of trunk muscle power

Trunk-muscle strength was measured using a device consisting of an iron frame combined with a QTM-06b [23]. Isometric trunk-muscle strengths in both extension and flexion were measured as the peak torque (Nm) with the maximum performance of pushing the pad of the measuring device. This torque-value was adjusted for the body weight (Nm/kg).

### Assessment of magnetic resonance imaging

A 1.5-Tesla superconducting imager and phased array coils was used for all subjects in the present study. A mobile magnetic resonance imaging unit (Intera Achieva 1.5-Tesla; Philips, Amsterdam, Nederland) was used. The cervical spine magnetic resonance imaging was performed with participants in the supine position. The imaging protocol included sagittal T2-weighted fast-spin echo (repetition time, 4000 ms/echo; echo time, 200 ms; field of view, 300 × 320 mm) and axial T2-weighted fast-spin echo (repetition time, 4000 ms/echo; echo time, 120 ms; field of view, 180 × 180 mm). Sagittal T2-weighted images were used to assess the intervertebral space from C2/3 to C7/T1.

### Evaluation of intervertebral disc degeneration on cervical magnetic resonance imaging

Intervertebral disc degeneration was evaluated based on the following two magnetic resonance imaging findings: (1) decrease in the signal intensity of the intervertebral discs, and (2) disc space narrowing. The cervical intervertebral levels evaluated for disc degeneration included all levels from C3/4 to C7/T1. To rate the magnetic resonance findings, we used Matsumoto’s classification [5]. The scoring system for the different magnetic resonance findings was as follows; decrease in signal intensity of intervertebral disc; grade 0: as hyperintense as or slightly less bright than cerebrospinal fluid, grade 1: markedly hypointense than cerebrospinal fluid, and grade 2: no signal. Disc space narrowing; grade 0: 100–75% of height of upper healthy disc, grade 1: 75–50% of height of upper healthy disc, and grade 2: less than 50% of height of upper healthy disc.

**Fig. 1:**
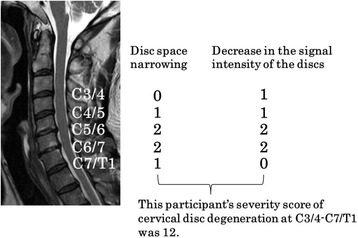
Evaluation of severity score of cervical spondylosis. Intervertebral disc degeneration was evaluated based on the following two magnetic resonance findings: (1) decrease in the signal intensity of the intervertebral discs, and (2) disc space narrowing. The cervical intervertebral levels evaluated for disc degeneration included all levels from C3/4 to C7/T1. To rate the magnetic resonance findings, we used Matsumoto’s classification [Matsumoto M, JBJS-Br 1998]

In addition, to determine the severity of cervical disc degeneration, the examiners summed the values of Matsumoto’s classification in each of the intervertebral sections. This summed value was defined as the severity score of cervical disc degeneration. According to this severity score, 0 corresponded to normal cervical disc and 20 to most severe degenerative cervical disc (Fig. 1).

In our preliminary data from 50 adults (mean age: 55.9 ± 16.6 years old), the intra-and inter-reliabilities of evaluating the decrease in signal intensity of the intervertebral discs/disc space narrowing on cervical magnetic resonance imaging were as follows: intra-observer agreements for WK (spine surgeon with 19-year experience) and OS (orthopedic surgeon with 5-year experience) were excellent (kappa = 0.839–0.913), and inter-observer agreements were moderate (kappa = 0.555–0.586).

### Measurement of anterior-posterior diameter of the cerebrospinal fluid column on mid-sagittal magnetic resonance imaging

**Fig. 2:**
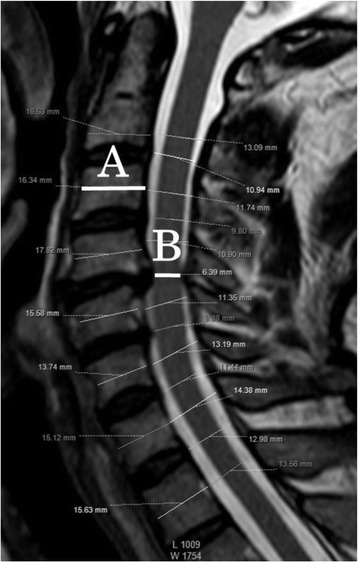
Measurement of the diameter on mid-sagittal magnetic resonance imaging. Minimum anterior-posterior diameter on mid-sagittal magnetic resonance imaging / anterior-posterior diameter of C3 vertebral body was calculated by dividing B by A as illustrated in this figure. A is the anterior-posterior diameter of C3 vertebral body, B is the minimum anterior-posterior diameter of spinal canal from C3/4 to C7/T1

We used a mid-sagittal magnetic resonance imaging scan to measure the anterior-posterior diameter of the cerebrospinal fluid column at the C3/4, C4/5, C5/6, C6/7, and C7/T1 intervertebral levels. In addition, the value obtained by dividing the anterior-posterior diameter of the cerebrospinal column by the mid-sagittal anterior-posterior diameter of C3 vertebral body was defined as % anterior-posterior diameter (Fig. 2).

The inter- and intra-observer agreements of cerebrospinal fluid column measurement from 50 adults were as follows: intra-class correlation coefficient [ICC (1,1)] for WK was 0.734 (95% confidence interval (CI): 0.575–0.839; *P* ≤ 0.001), and for OS was 0.818 (95% CI: 0.680–0.896; *P* ≤ 0.001). Interclass correlation coefficient [ICC (2,1)] in WK and OS was 0.623 (95% CI: 0.413–0.768; *P* ≤ 0.001).

### Assessment of bone status, serum bone metabolic markers and essential amino acids

Dual energy X-ray absorptiometry of forearm was measured and the bone mineral density scores were calculated as T-score. Blood samples were collected in the early morning, and these were immediately centrifuged. Serum samples were extracted and transferred to a deep freezer. Serum bone-specific alkaline phosphatase (μg/dL) was quantified using a chemiluminescent enzyme immunoassay/antibody radioimmunoassay (LSI Medience Corp., Tokyo, Japan). Serum pentosidine concentration (pmol/mL, high performance liquid chromatography; LSI Medience Corp., Tokyo, Japan), undercarboxylated osteocalcin (ng/mL,:electro-chemiluminescence immunoassay; LSI Medience Corp., Tokyo, Japan), cross-linked N-telopeptide of type 1 collagen (nMBCE/L, enzyme immunoassay; LSI Medience Corp., Tokyo, Japan), bone tartrate-resistant acid phosphatase-5b (mU/dL, enzyme immunoassay; LSI Medience Corp., Tokyo, Japan), total type 1 procollagen N-terminal propeptide (μg/dL, electro-chemiluminescence immunoassay; LSI Medience Corp., Tokyo, Japan), 25(OH)D3 (ng/mL, liquid chromatography-tandem mass spectrometry; LSI Medience Corp., Tokyo, Japan), and essential amino acids (tryptophan, lysine, methionine, threonine, valine, leucine, isoleucine, and histidine; nmol/mL, high performance liquid chromatography) were measured.

### Statistical analysis

The data were analyzed using SPSS ver. 22.0 (SPSS Inc., Chicago, IL, USA). All data were grouped according to sex.

For multiple group comparisons of % anterior-posterior diameters and disc degenerative scores between age groups, we used analysis of variance. Statistical significance of individual differences was evaluated using the Tukey’s honestly significant difference test if analysis of variance was significant. The % anterior-posterior diameter and the disc degenerative score were analyzed for each disc level.

Spearman correlation coefficient was used to evaluate whether % anterior-posterior diameter at each level was correlated with the severity score of cervical disc degeneration.

For correlations between the severity score of cervical disc degeneration and other parameters: age, body mass index, skeletal muscle index, exercise habit, T score of bone mineral density, and the serum values (bone-specific alkaline phosphatase, pentosidine, cross-linked N-telopeptide of type 1 collagen, bone tartrate-resistant acid phosphatase-5b, 25(OH)D3, isoleucine, threonine, tryptophan, valine, histidine, phenylalanine, methionine, lysine, and leucine); Spearman correlation coefficient was used. We conducted stepwise multiple regression analyses with the severity score of cervical disc degeneration as the dependent variable and parameters showing significant correlation by single correlation analysis as independent variables.

*P* values < 0.05 were considered statistically significant.

## Results

### Comparison of % anterior-posterior diameters and disc degenerative scores between age groups

**Fig. 3:**
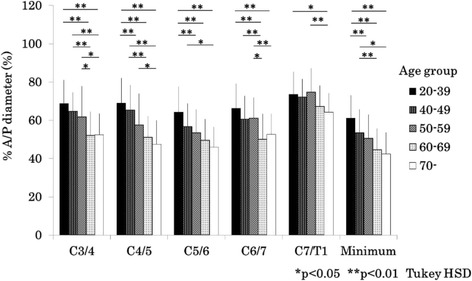
Bar chart (mean and standard deviation) showing the % anterior-posterior diameter by age at each level and minimum % anterior-posterior diameter in men. A/P: anterior-posterior, HSD: honestly significant difference

**Fig. 4:**
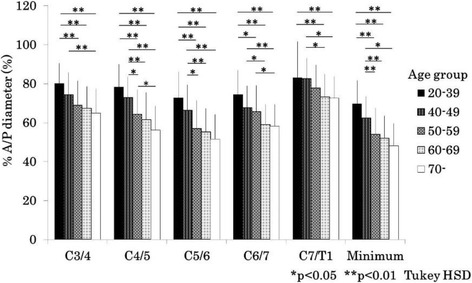
Bar chart (mean and standard deviation) showing the % anterior-posterior diameter by age at each level and minimum % anterior-posterior diameter in women. A/P: anterior-posterior, HSD: honestly significant difference

**Fig. 5:**
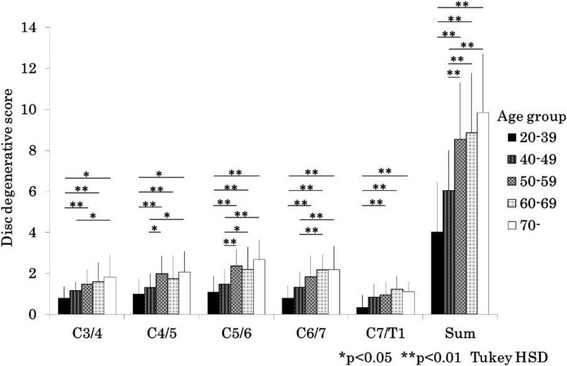
Bar chart (mean and standard deviation) showing disc degenerative score by age at each level and sum of score in men. HSD: honestly significant difference

**Fig. 6:**
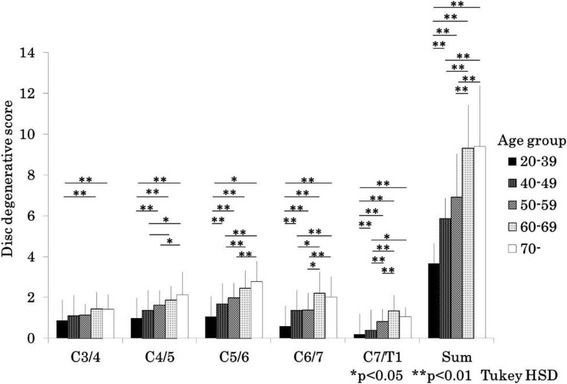
Bar chart showing (mean and standard deviation) the disc degenerative score by age at each level and sum of score in women. HSD: honestly significant difference

In men, % anterior-posterior diameter was significantly reduced with aging at the levels of C3/4, C4/5, C5/6, C6/7 and minimum diameter from the 20s and 30s, to 60s age groups (Fig. 3). Almost the same trend was observed in women (Fig. 4). The disc degeneration score increased significantly as the participants became older for both sexes. Severity score of the disc degeneration was doubled in the 50–59 years group as compared to that in the 20–39 age group in men (Fig. 5) and women (Fig. 6). In both sexes, % anterior-posterior diameter was the smallest, and disc degenerative score was the lowest at C5/6.

### Correlation of spinal canal diameter with disc degeneration score

There was a significant negative correlation between % anterior-posterior diameters and disc degenerative scores at all levels in both sexes. Values of correlation coefficients ranged from − 0.646 to − 0.325 in the same intervertebral levels. Values of correlation coefficients between the diameter in each intervertebral level and the severity score of cervical disc degeneration ranged from − 0.376 to − 0.625. The correlation between the minimum % anterior-posterior diameter and the severity score of cervical disc degeneration was − 0.593 in men and − 0.527 in women (Tables 2 and 3).

**Table 2:** Spearman correlation of parameters in 151 men

Caracterics	Age	Minimum % A/P diameter	Sevierity score of CDD	BMI	SMI	TMS in flexion	TMS in extention	Exercise habit	T score of BMD	BAP	NTX	TRACP-5b	total P1NP	25(OH)D3	Pent	Thr	Trp	Val	His	Phe	Met	Ile	Lys	Leu
Age		-.515^**^	.637^**^	−.023	−.121	-.534^**^	-.271^**^	.100	-.395^**^	.000	-.298^**^	.122	-.281^**^	.284^**^	.586^**^	.016	-.230^**^	−.105	-.235^**^	.095	−.036	−.103	.020	-.252^**^
Minimum % A/P diameter			-.593^**^	−.088	−.073	.261^**^	.104	−.035	.001	.015	.231^**^	−.104	.149	-.175*	-.384^**^	.016	.112	−.095	.007	.005	−.041	.056	−.048	−.016
Sevierity score of CDD				−.017	−.006	-.293^**^	−.131	−.050	−.151	.014	-.325^**^	.076	-.164^*^	.135	.338^**^	−.037	-.172^*^	−.156	-.178^*^	−.114	−.066	-.192^*^	−.032	-.230^**^
BMI					.874^**^	.253^**^	.417^**^	.033	.099	−.127	−.144	-.281^**^	−.133	.015	−.109	−.011	−.081	.163^**^	.062	.163^*^	−.050	.087	−.015	.107
SMI						.234**	.425**	.024	.213^**^	−.059	−.090	-.220^**^	−.067	.032	−.149	−.074	−.065	.121	.055	.096	−.091	.041	−.005	.092
TMS in flexion							.490**	−.168	.226^*^	−.108	.022	−.130	.072	−.139	-.377**	−.021	.128	.096	.146	.012	.040	.152	−.018	.176
TMS in extention								−.140	.288^**^	−.068	−.088	−.067	.061	−.050	-.220^*^	.046	−.017	−.025	.127	−.053	−.030	−.028	−.097	.038
Exercise habit									.003	−.026	−.027	−.024	.043	−.102	.112	−.076	−.109	.129	−.104	.026	−.007	.081	−.042	.101
T score of BMD										-.262^**^	−.081	−.075	−.102	−.070	-.162^*^	−.015	−.055	.037	.173^*^	−.077	.032	.011	−.041	.129
BAP											.370^**^	.522^**^	.639^**^	.040	−.017	.010	.153	.057	−.028	−.006	.095	.074	.006	.073
NTX												.360^**^	.481^**^	−.129	−.140	−.053	.088	.042	−.010	−.081	−.017	.111	.037	.135
TRACP-5b													.502^**^	.098	.113	−.060	.035	−.066	−.148	−.154	−.002	−.002	−.055	−.030
total P1NP														−.025	−.152	.060	.120	.002	.077	−.039	.102	.038	.028	.093
25(OH)D3															.252**	.004	−.069	−.038	.049	.179*	.116	−.137	.169*	−.057
Pent																−.030	−.096	.028	−.080	.216**	.114	−.031	.117	−.095
Thr																	.246^**^	.056	.385^**^	.215^**^	.660^**^	.069	.402^**^	.027
Trp																		.383^**^	.283^**^	.344^**^	.493^**^	.414^**^	.293^**^	.484^**^
Val																			.169^*^	.366^**^	.321^**^	.842^**^	.291^**^	.892^**^
His																				.297^**^	.530^**^	.049	.362^**^	.243^**^
Phe																					.546^**^	.299^**^	.508^**^	.379^**^
Met																						.335^**^	.626^**^	.381^**^
Ile																							.200^*^	.873^**^
Lys																								.319^**^
Leu																								

*p < 0.05, **P < 0.0001

*Abbreviations*: *A/P* anterior-posterior, *Sevierity score of CDD* severity score of cervical disc degeneration from C3/4 to C7/T1, *BMI* body mass index, *SMI* Skeletal muscle mass index, *TMS* trunk muscle strength, *BMD* bone mineral density, *BAP* bone-specific alkaline phosphatase, *NTX* cross-linked N-telopeptide of type 1 collagen, *TRACP-5b* bone tartrate-resistant acid phosphatase-5b, *total P1NP* total type 1 procollagen N-terminal propeptide, *Pent* pentosidine, *Thr* threonine, *Trp* tryptophan, *Val* valine, *His* histidine, *Phe* phenylalanine, *Met* methionine, *Ile* isoleuicine, *Lys* lysine, *Leu* leucine

**Table 3:** Spearman correlation of parameters in 193 women

Caracterics	Age	Minimum % A/P diameter	Sevierity score of CDD	BMI	SMI	TMS in flexion	TMS in extention	Exercise habit	T score of BMD	BAP	NTX	TRACP-5b	total P1NP	25(OH)D3	Pent	Thr	Trp	Val	His	Phe	Met	Ile	Lys	Leu
Age		-.516^**^	.683^**^	.266^**^	.227^**^	-.432^**^	-.171^*^	.179^*^	-.776^**^	.455^**^	.305^**^	.585^**^	.196**	.507^**^	.542^**^	−.127	-.221^**^	−.016	−.019	.139	−.059	−.127	.254^**^	−.127
Minimum % A/P diameter			-.527^**^	-.226^**^	-.173^*^	.208^**^	−.011	−.048	.411^**^	-.364^**^	-.214^**^	-.358^**^	-.190^**^	-.280^**^	-.233^**^	.061	.099	−.086	−.043	−.075	−.002	−.011	-.162^*^	−.021
Sevierity score of CDD				.287^**^	.243**	-.268^**^	−.059	.005	-.533^**^	.399^**^	.227^**^	.470^**^	.253^**^	.342^**^	.345^**^	−.065	−.091	.039	.009	.080	−.070	−.109	.322^**^	−.056
BMI					.800^**^	−.097	.011	.072	-.210^**^	.184^*^	.101	.092	.069	.176^*^	−.141	.153^*^	.011	.248^**^	.145^*^	.137	.011	.177^*^	.129	.144^*^
SMI						−.085	.041	.031	−.124	.161^*^	.048	.114	.090	.172^*^	−.114	.161^*^	−.063	.172^*^	.141	.145^*^	.081	.115	.124	.143^*^
TMS in flexion							.458^**^	.035	.344^**^	-.191^*^	-.174^*^	-.240^**^	.029	-.193^*^	-.287^**^	.054	.112	.074	.114	.026	.071	.033	−.090	.120
TMS in extention								.098	.178^*^	−.006	−.028	−.037	.058	−.009	-.188^*^	.075	.109	.150	.103	.047	.026	.039	−.026	.158^*^
Exercise habit									-.145^*^	.033	−.013	.022	−.046	.143^*^	.096	−.044	−.120	.066	.038	−.002	−.061	.024	−.016	.050
T score of BMD										-.547^**^	-.412^**^	-.586^**^	-.348^**^	-.419^**^	-.372^**^	.057	.153^*^	−.041	.069	−.115	.011	.035	-.261^**^	.068
BAP											.535^**^	.777^**^	.713^**^	.255^**^	.130	−.067	.017	.106	.040	.116	.058	.075	.267^**^	.090
NTX												.679^**^	.576^**^	.124	.143^*^	.032	−.068	−.002	.043	.118	.039	.061	.205^**^	.023
TRACP-5b													.697^**^	.342**	.327^**^	−.002	−.076	−.018	−.014	.065	.030	−.048	.290^**^	−.028
total P1NP														.083	.038	.035	−.010	.081	.002	.055	.057	.052	.229^**^	.074
25(OH)D3															.410^**^	−.073	−.082	.055	.144^*^	.087	−.024	−.104	.245^**^	−.005
Pent																-.190^**^	−.139	-.227^**^	.012	.130	−.037	-.201^**^	.101	-.221^**^
Thr																	.198^**^	.194^**^	.377^**^	.118	.609^**^	.203^**^	.467^**^	.179^*^
Trp																		.408^**^	.278^**^	.244^**^	.337^**^	.355^**^	.233^**^	.420^**^
Val																			.238^**^	.282^**^	.264^**^	.768^**^	.287^**^	.866^**^
His																				.332^**^	.444^**^	.214^**^	.473^**^	.413^**^
Phe																					.484^**^	.329^**^	.279^**^	.382^**^
Met																						.380^**^	.480^**^	.369^**^
Ile																							.176^*^	.819^**^
Lys																								.297^**^
Leu																								

*p < 0.05, **P < 0.0001

*Abbreviations*: *A/P* anterior-posterior, *Sevierity score of CDD* severity score of cervical disc degeneration from C3/4 to C7/T1, *BMI* body mass index, *SMI* Skeletal muscle mass index, *TMS* trunk muscle strength, *BMD* bone mineral density, *BAP* bone-specific alkaline phosphatase, *NTX* cross-linked N-telopeptide of type 1 collagen, *TRACP-5b* bone tartrate-resistant acid phosphatase-5b, *total P1NP* total type 1 procollagen N-terminal propeptide, *Pent* pentosidine, *Thr* threonine, *Trp* tryptophan, *Val* valine, *His* histidine, *Phe* phenylalanine, *Met* methionine, *Ile* isoleuicine, *Lys* lysine, *Leu* leucine

### Correlation of the cervical disc degeneration with physical, bone status and biochemical markers, and multiple regression models for elucidating the factors correlated with the cervical disc degeneration on magnetic resonance imaging

The severity score of the cervical disc degeneration was significantly correlated with age, trunk-muscle strength in flexion, pentosidine, cross-linked N-telopeptide of type 1 collagen, and leucine in men (Table 2); and with age, body mass index, skeletal muscle index, trunk-muscle strength in flexion, T score of bone mineral density, pentosidine, bone tartrate-resistant acid phosphatase-5b, cross-linked N-telopeptide of type 1 collagen, undercarboxylated osteocalcin, 25(OH)D3, lysine in women (Table 3).

In multiple linear regression analysis, age, cross-linked N-telopeptide of type 1 collagen and isoleucine were significantly correlated with the total cervical degeneration score in men (R^2^ = 0.470, Table 4), and age and lysine were significantly correlated with the total cervical degeneration score in women (R^2^ = 0.500, Table 5).

**Table 4:** Analysis of the factors correlated with the severity score of cervical disc degeneration on magnetic resonance imaging in men

R^2^ = 0.470	B	β	P value	95% CI
Age	0.119	0.557	< 0.001	0.089 to 0.149
NTX	−0.094	−0.156	0.027	−0.177 to −0.011
Isoleucine	− 0.045	− 0.181	0.008	− 0.078 to − 0.012

*Abbreviations*: *CI* confidence interval, *NTX* cross-linked N-telopeptide of type 1 collagen

**Table 5:** Analysis of the factors correlated with the severity score of cervical disc degeneration on magnetic resonance imaging in women

R^2^ = 0.500	B	β	*P* value	95% CI
Age	0.147	0.658	< 0.001	0.121 to 0.173
Lysine	0.015	0.136	0.023	0.002 to 0.027

*Abbreviation*: *CI* confidence interval

## Discussion

The present cross-sectional study evaluated the correlation between cervical canal stenosis and degeneration of the intervertebral disc in the general population, and analyzed factors related to cervical intervertebral disc degeneration. With aging, the cervical canal diameters became smaller and the cervical disc degeneration scores became higher. Kato et al. reported that spinal dural tube diameter tended to decrease with age, especially at the intervertebral disc levels, and showed that degenerative changes in the cervical spine progressed chiefly in the intervertebral discs [8]. In a previous report, prevalence of cervical cord compression was higher in men than in women [7]. In the current study, spinal canal anterior-posterior diameter normalized by C3 vertebral body was larger in women than in men. In addition, the smallest relative diameter was at C5/6, followed by C4/5 and C6/7. This was in line with previous reports showing that spinal cord compression was most frequent at C5/6 [7, 24].

A significant negative correlation was found between % anterior-posterior diameters and disc degeneration score at all levels in both sexes. Morishita et al. reported that disc degeneration grades were worse in symptomatic subjects with two-level cervical segments stenosis than in those with one-level stenosis [25]. They proposed that multi-level cervical stenosis may develop following initial stenosis at the C5/6 segment. In this study, disc degeneration score at C5/6 was the most advanced in both sexes.

**Fig. 7:**
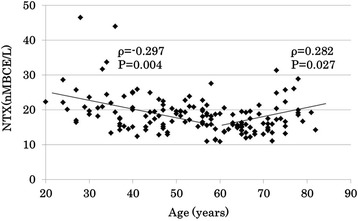
Scatter plot showing the correlation between age and cross-linked N-telopeptide of type 1 collagen in men. NTX: cross-linked N-telopeptide of type 1 collagen

In Spearman correlation coefficient, age was moderately correlated with the severity score of cervical disc degeneration in both sexes, age explained > 40% of the variance in men and > 45% in women. The other factors explained from 2 to 28%. Omair reported that age was the most significant determinant of lumbar disc degeneration in their genetic association study [26]. Age might also be the strongest factor related to cervical disc degeneration in our current study. In multiple linear regression analysis, age, cross-linked N-telopeptide of type 1 collagen and isoleucine were significantly correlated with the total cervical degeneration score in men (R^2^ = 0.470, Table 4), while age and lysine were significantly correlated with the total cervical degenerative score in women (R^2^ = 0.500, Table 5). Goode reported a positive correlation, adjusted for sex, between severity of lumber disc space narrowing and urinary cross-linked N-telopeptide of type 1 collagen [19]. Thoracic spinal disc space narrowing was one of the risk factors for vertebral fractures in men over 50 years old [27]. Only a few studies reported the relation between bone turnover and osteoporosis in men, and Goode’s data were opposite to this survey, which showed a negative correlation between spinal disc degeneration and serum cross-linked N-telopeptide of type 1 collagen. However, these previous reports did not look at the cervical spine specifically. In the present study, there was a significant negative correlation between age and cross-linked N-telopeptide of type 1 collagen under 60 years of age (ρ = − 0.297, *P* = 0.004), while there was a positive correlation over 60 years of age (ρ = 0.282, *P* = 0.027) in men (Fig. 7). This trend was similar to the longitudinal data reported by Yoshimura et al. Furthermore, disc degenerations were more severe at 50–59 years of age than below 50 years, and there was no difference between 50 and 59 years of age and over 60 years. There was a significant correlation between disc degenerative score and age, isoleucine, tryptophan, valine, and leucine below 60 years of age. Over 60 years of age, only 25(OH) D3 was significantly correlated with disc degeneration scores (Table 6). Factors related to disc degeneration may differ according to age.

**Table 6:** Correlation coefficients between disc degenerative score and parameters in men under 60 years old and over 60 years old

Age	parameters	ρ	P value
Under 60 years	Age	0.668	< 0.001
	NTX	−0.380	< 0.001
	Isoleucine	−0.352	0.001
	Tryptophan	−0.252	0.017
	Valine	−0.255	0.033
	Leucine	−0.303	0.004
Over 60 years	25(OH) D3	−0.302	0.017

*Abbreviation*: *NTX* cross-linked N-telopeptide of type 1 collagen

Isoleucine, valine and leucine are branched chain amino acids. Branched chain amino acids are one of the disc components [21]. Leucine intake was associated with long-term lean body mass retention [28]. Disc degeneration was positively correlated with paraspinal muscle atrophy and fatty infiltration [16, 17], and muscle strength had a greater impact on disc degeneration [29] at the lumbar spine. Okada reported that there was no significant correlation between cervical disc degeneration and cross-sectional area of the posterior cervical extensor muscles [14]. In the current study, trunk muscle strength in flexion was associated with cervical disc degeneration in univariate correlation. Trunk muscles strength may be related to cervical disc degeneration. Polymorphisms were found in the gene encoding vitamin D receptor [30, 31]. A longitudinal study is required to clarify whether there is a relation between the ingestion of branched chain amino acids and increasing bone turnover in individuals younger than 60 years, and whether ingestion of vitamin D3 in those over 60 years of age can prevent progression of disc degeneration. In women, lysine may be a biomarker for disc degeneration. In human thoracic discs, the content of pyridinoline decreased with increasing age [32], and lysine was associated with collagen cross-linkage [33]. The association between serum lysine and collagen cross-linking metabolites remain unclear. Longitudinal research is necessary to clarify the usefulness of lysine as a biomarker for disc degeneration.

The present study has several limitations. First, it was a cross sectional study, and therefore, the rate of progression of disc degeneration could not be clarified. Second, our survey was limited to Japanese subjects, and hence possible ethnic differences were not considered. Third, in our participants the proportion of smoking habits (men: 60.9% current and ex-smokers, 21.9% current smokers; women: 20.7% current and ex-smokers, 9.3% current smokers) may be lower than that in the general population as is the prevalence of diabetes (men: 7.9%, women: 4.6%). The subjects of this survey included those receiving osteoporosis treatment (men: 0.7%, women: 4.1%). These conditions may affect disc degeneration and/or bone metabolism. Fourthly, qualitative assessment of disc space narrowing might be difficult in patients with multiple vertebral disc reductions. However, there was an acceptable validation for the evaluation of the disc degeneration on MRI in this study; therefore this limitation is not expected to have a major impact on the analysis.

## Conclusions

The anteroposterior diameters of cervical spine on magnetic resonance imaging decreased with age in both sexes. The anteroposterior diameters were correlated to disc degeneration. In multiple linear regression analysis, age, cross-linked N-telopeptide of type 1 collagen and isoleucine were significantly correlated with the cervical disc degenerative score in men, while age and lysine were significantly correlated with the degenerative score in women. Factors contributing to cervical disc degeneration were different between men and women. Whether modifying these significant factors is possible, or whether this intervention would contribute to prevention of disc degeneration requires future studies.
